# Chemopreventive Potential of Synergy1 and Soybean in Reducing Azoxymethane-Induced Aberrant Crypt Foci in Fisher 344 Male Rats

**DOI:** 10.1155/2011/983038

**Published:** 2011-02-22

**Authors:** V. P. Gourineni, M. Verghese, J. Boateng, L. Shackelford, K. N Bhat

**Affiliations:** ^1^Nutritional Biochemistry and Carcinogenesis Laboratory, Department of Food and Animal Sciences, Alabama A&M University, P.O. Box 1628, Normal, AL 35762, USA; ^2^Department of Chemistry, Alabama A&M University, Normal, AL 35762, USA

## Abstract

Synergy1, a prebiotic composed of Inulin and Oligofructose (1 : 1). Soybean meal is a natural source of isoflavones. The objective was to investigate the effects of feeding Synergy1 and SM on the incidence of azoxymethane- (AOM-) induced aberrant crypt foci (ACF) in Fisher 344 male rats. Rats (54) were randomly assigned to 9 groups (*n* = 6). Control group (C) was fed AIN-93G and treatment groups Syn1 and SM at 5% and 10% singly and in combinations. Rats were injected with two s/c injections of AOM at 7 and 8 weeks of age at 16 mg/kg body weight and killed at 17 weeks by CO_2_ asphyxiation. Colonic ACF enumeration and hepatic enzyme activities were measured. Reductions (%) in total ACF among treatment groups fed combinations were higher (67–77) compared to groups fed singly (52–64). Synergistic mechanisms among phytochemicals may be responsible suggesting protective role in colon carcinogenesis with implications in food product development.

## 1. Introduction

Cancer is a major public health problem and statistics show that colon cancer is the third leading cause of cancer deaths among men and women in the USA [1]. Primary prevention is a promising and cost-effective approach in reducing morbidity and mortality related to cancer. Geographical variations in colon cancer prevalence are higher in industrialized nations compared to developing countries which may be explained by the influence of environmental factors such as diet [2]. Diet is one of the modifiable risk factors which has been found to influence colonic microflora and their enzymes in colon carcinogenesis [3–5]. Dietary chemoprevention emerged as one of the strategies targeting multistage pathogenesis [6]. Chemoprevention is defined as “use of natural, synthetic, biological or chemical agents to reverse, suppress, or prevent carcinogenic progression [7–9].

Epidemiological and experimental studies show a correlation with the consumption of dietary fiber and a reduction in colon cancer [10–12]. Synergy1 is a prebiotic composed of Inulin and Oligofructose in equal proportion that has shown inhibitory effects on AOM-induced ACF in F344 rats [13] explained by stimulation of probiotics, production of short chain fatty acids, and activation of detoxifying enzymes [14].

Soybean contains a variety of phytochemicals such as isoflavones, phytates, phytosterols, protease inhibitors, acid phenolics, saponins, and omega-3 fatty acids with numerous health benefits such as lowering plasma cholesterol, osteoporosis, prostate, and breast cancer [15, 16]. Isoflavones are phytoestrogens available as glycosides in plants and are transformed to aglycones in the human intestine. Action of intestinal *β*-glucosidases results in increased absorption and bioavailability of these bioactive compounds and formation of metabolites such as equol, O-desmethylangolensin (ODMA) from daidzein, and P-ethylphenol from genistein [17–21]. The human population consists of equol producers and nonequol producers based on interindividual differences in establishment of intestinal microflora [22]. Due to its potential antioxidative properties [23, 24] equol has been reported to reduce the development of chronic diseases such as prostate cancer [25] and cardiovascular diseases [26]. 

Biomarkers aid in the screening and diagnosis of early stages of pathogenesis [27]. One such early biomarker in colon carcinogenesis is Aberrant Crypt Foci (ACF)—used in prescreening the effects of potential chemopreventive agents and chemical risk assessment in the environment. ACF are microscopic precancerous lesions in the colon capable of progressing to tumors [28]. Azoxymethane (AOM) is a potent carcinogen used to induce colon tumors in rodent models to determine the chemopreventive efficacy of foods [29].

Quantitative assays of specific enzymes activated in response to xenobiotics are useful biomarkers in determining the chemopreventive potential of specific diets [30, 31]. Glutathione-S-transferases (GSTs) are crucial phase II detoxification enzymes that offer protection by catalyzing conjugation resulting in excretion of xenobiotics [32, 33]. Catalase is an antioxidative enzyme involved in the detoxification of hydrogen peroxide. Superoxide-dismutase is an essential enzyme involved in the conversion of free radical superoxides to peroxides [34]. Prebiotics may influence isoflavone metabolism that can constitute a novel approach in inducing beneficial microflora such as equol-producing and lactic acid bacteria responsible for healthy bowel function. Previous research on health benefits of Synergy1 and Soybean has provided a platform to investigate the effects of Synergy1 and Soybean meal (SM) singly and in combinations at 5% and 10% on the incidence of aberrant crypt foci (ACF) in Fisher 344 male rats and explore the selected mechanisms of action.

## 2. Methods and Materials

### 2.1. Animal Housing and Diets

 Fifty-four Fisher 344 male weanling rats (3 weeks old) were obtained from Harlan, IN and housed in stainless steel wire cages at 2 rats per cage. After one week of acclimatization all rats were randomly assigned to nine groups (*n* = 6) and fed the following diets: AIN-93G as control (C) [35] and treatment groups with C+ Synergy1 (5%, 10%), C+ SM (5%, 10%), C+ Synergy1 + SM (5% + 5%), (10% + 10%), (5% + 10%), and (10% + 5%). Dietary modifications were made to fiber, casein, and cornstarch (Table 1). All rats were housed and maintained according to standard protocols. Biweekly body weights and daily feed intakes were recorded. The diets were prepared fresh and stored at 4°C. Dietary ingredients were obtained from MP Biomedicals, Costa Mesa, CA. Synergy1 (Beneo) was obtained from Orafti (Teinen, Belgium) and SM was obtained from the local market (Garden Cove, Huntsville, AL). The Institutional Animal Care and Use Committee of Alabama A&M University approved all protocols involving the experiment.

### 2.2. Carcinogen Injection

All rats except saline controls received two subcutaneous injections of AOM in saline (National Chemical Repository, Kansas City, MO) for ACF induction at 16 mg/kg body at 7 and 8 weeks of age. At 17 weeks of age, rats were killed using CO_2_ asphyxiation following an overnight fast.

### 2.3. Sample Collection

The cecum from each rat was excised, weighed and split open, and the pH of the cecal content was measured. Livers of rats were excised and stored at −80°C until analysis.

### 2.4. Enumeration of Aberrant Crypt Foci (ACF)

Aberrant crypt foci scoring (ACF) was done as described by Bird [36]. Briefly, excised colons were flushed with phosphate buffer solution (0.1 M, pH 7.2) (Fisher Scientific, Suwannee, GA) fixed on filter paper with 10% buffered formalin (Fisher Scientific, Suwannee, GA). Fixed colons were sectioned into proximal (closer to the cecum) and distal portions (closer to the rectal end) of equal length. The colon segments were stained with a 0.2% methylene blue solution (Sigma chemicals, St. Louis, MO), and ACF were scored microscopically.

### 2.5. Preparation of Liver Samples for Measuring Hepatic Enzyme Activity

One gram of liver sample was homogenized in 10 mL of potassium phosphate buffer (pH 7.2, 0.1 M) using a Potter-Elvehjem homogenizer (Model 985370-395, Fisher Scientific, Suwannee, GA). The homogenate was centrifuged (Eppendorf AG-5418, Fisher Scientific, Suwannee, GA) at 10,000 × g for 30 min and the supernatant was stored at 4°C.

### 2.6. Glutathione-S-Transferase (GST) Activity

The supernatant from liver samples was mixed with 1, chloro 2, 4-dinitrobenzene (1 mM), potassium phosphate buffer (0.1 M), and glutathione (1 mM). Sample (50–100 *μ*L) was analyzed using a Cary1/3 UV/VIS dual beam spectrophotometer (Varian, Palo Alto, CA) at 340 nm. The total enzyme activity was measured at the end of 5 min of reaction.

### 2.7. Reduced Glutathione (GSH) Assay

Hepatic GSH was estimated using Ellman's reagent [37]. An aliquot of the homogenate was deproteinized by the addition of an equal volume of 4% sulfosalicylic acid and centrifuged at 17,000 × g for 15 min at 2°C. To the diluted supernatant (0.5 mL), 4.5 mL of Ellman's reagent was added. Absorbance was read at 412 nm.

### 2.8. Catalase (CAT) Assay

Hepatic catalase activity was estimated at 240 nm by monitoring the decomposition of H_2_O_2_ [38]. The reaction mixture (1 mL) contained 0.02 mL of liver homogenate in phosphate buffer (50 mM, pH7.0) and 0.1 mL of 30 mM H_2_O_2_ in phosphate buffer.

### 2.9. Superoxide-Dismutase (SOD) Assay

Hepatic superoxide-dismutase was assayed as described by Fridovich [39]. To the supernatant (2.0 mL), 2.5 mL of 0.05 M carbonate buffer and 0.3 mM adrenaline were added and absorbance was measured at 480 nm.

### 2.10. Bone Mineralization

Specific minerals (Calcium (Ca), Phosphorus (P), Magnesium (Mg), Iron (Fe) and Zinc (Zn)) in femur bone samples were quantified using inductively coupled plasma spectroscopy (ICP) following standard protocol AACC Method [40].

### 2.11. Statistical Analysis

Data were analyzed (*n*: triplicates) using SAS 9.1 statistical program (SAS, Cary, NC). Results were analyzed by analysis of variance (ANOVA) and expressed as means ± SEM. Means were separated using Tukey's studentized range test. Significant differences (*P* < .05) were determined using one-way ANOVA.

## 3. Results and Discussion

### 3.1. Feed Intake, Weight Gain, Cecal Weight, and Cecal pH

There were no significant differences in feed intake (g) and weight gain (g) among groups (Table 2). Weight gain (278.3 g) was the highest in rats fed SM (5%). Cecal weight was significantly (*P* < .05) higher in treatment groups compared to rats fed the control diet. Rats fed Syn1 (10%) singly and Syn1 + SM (10%) in combination had significantly (*P* < .05) higher cecal weight compared to the other treatment groups (Table 2). Cecal pH ranged from a high of 7.40 in rats fed the control diet to a low of 5.99 in the rats fed Syn1 + SM (10%). Rats fed treatment diets excluding SM (5%) and SM (10%) had significantly (*P* < .05) lower cecal pH compared to the rats fed the control diet (Table 2). The groups fed Syn1 + SM (5 + 5%) and Syn1 + SM (10 + 5%) had the lowest cecal pH among groups fed combination diets.

### 3.2. Incidence of Aberrant Crypt Foci

**Figure 1:**
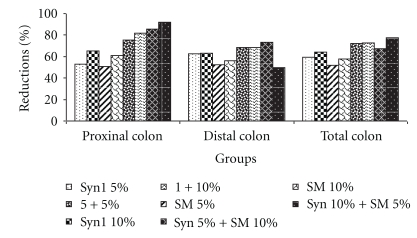
Reductions in azoxymethane-induced aberrant crypt foci compared to the control. Abbreviations used: Syn1: Synergy1 and SM: Soybean meal.

 Incidence of ACF in the distal colon was significantly (*P* < .05) higher compared to the proximal colon. These results are consistent with previous reported studies [41, 42]. Rats fed treatment diets had significantly (*P* < .05) lower number of ACF in the distal and proximal colon compared to the rats fed the control diet (Table 3). The rats fed the control diet had the highest number of ACF while the group fed Syn1 (10%) + SM (5%) had the lowest number of ACF with a 77.3% reduction, compared to the control. Total ACF (Table 3) ranged from a high of 150 in the control rats to a low of 34 in the rats fed Syn1 (10) + SM (5%). Reductions in total ACF compared to the control in rats fed the treatment diets ranged from a high of 77.3% in the group fed Syn1 (10) + SM (5%) to a low of 52% in the group fed SM (5%) (Figure 1). The groups fed the combination diet with higher concentrations of Syn1 (10%) + SM (10%) had the greatest reductions in ACF. Feeding Syn1 and SM alone at 5% and 10% resulted in lower reductions of 59, 64, 52 and 57.6%, respectively, compared to the control. However, when Syn1 was fed in combination with SM at both 5% and 10% levels, the reductions were greatly enhanced (75–91% in the proximal and 49–73% in the distal colon compared to the control fed group).

### 3.3. Total Crypts

Total colonic crypts are precise indicators of the chemopreventive effect of a nutrient or food since it takes into account both the ACF as well as crypts per focus. Total crypts were significantly (*P* < .05) higher in the rats fed the control compared to those fed treatment diets. Rats fed Syn1 + SM (5%) and Syn1 + SM (10%), Syn1 (10%) + SM (5%) and Syn1 (5%) + SM (10%) had significantly (*P* < .05) lower number of total crypts compared to the other groups fed treatment diets. Total crypts ranged from a high of 440 in the control rats to a low of 99 in the rats fed Syn1 (10%) + SM (5%). The reduction in total crypts was enhanced with the addition of SM to Syn1 (Table 4). The reductions in total crypts compared to the control ranged from a low of 49.54% in the group fed SM (5%) to a high of 77.70% in the rats fed Syn1 (10%) + SM (5%).

### 3.4. Hepatic GST, GSH, CAT, and SOD

GST activity (*μ*mol/mg) in the treatment groups was significantly (*P* < .05) higher (21.68 to 26.77 *μ*mol/mg) compared to the control group (10.58 *μ*mol/mg) (Table 5). GST activity in the treatment groups was 51 to 60% higher compared to the control. A similar trend was observed with GSH levels; however GSH levels (mM) were over 90% higher in the treatment groups compared to the control (Table 5). Catalase (CAT) activity (*μ*mol/mL) in the liver of rats fed the control diet (0.055) (Table 5) was significantly (*P* < .05) lower compared to the treatment groups. Rats fed treatment diets had 51 to 80% higher CAT activity (*μ*mol/mL) compared to those fed the control diet. Among the rats fed treatments diets, CAT activity was the highest in the rats fed Syn1 + SM (10%) (0.330) which accounted for an increase of 83% compared to the control, and the lowest activity was seen in the rats fed SM (10%) (0.112) accounting for an increase of 51% compared to the control. There were no differences in CAT activity among treatment groups. SOD activity (*μ*mol/mL) did not differ among rats fed control and treatment diets. However, rats fed the control diet had a lower (0.107) SOD activity (*μ*mol/mL) compared to those fed treatment diets (0.176 to 0.190).

### 3.5. Bone Mineralization

Specific minerals in bone femurs (Ca, P, Mg, Fe, and Zn) were significantly (*P* < .05) higher in treatment groups compared to control (Table 6). Among treatment groups, rats fed Syn1 + SM (10% + 10%) showed the highest retention of these minerals. Calcium (mg/g) retention in rats fed treatment diets in combinations was over twice compared to the control. Calcium and phosphorus form major composition of bone and involved in its remodeling process.

## 4. Discussion

The ACF model has been used [43–46] to study dietary modulation of colon carcinogenesis. Weight gain and feed intakes among control and treatment groups were similar. Average feed intake ranged from *≈*16 to 18 g/day. Feeding functional foods or dietary ingredients has been reported to affect weight gain in some published studies [47]. However, other researchers [48] did not observe any differences in weight gain or feed intake. Rats fed diets containing Syn1 (10%) singly and in combination with Soybean meal had significantly (*P* < .05) lower cecal pH and higher cecal weight which is consistent with previous studies [49, 50]. The fermentation of soluble fiber by colonic microflora results in the production of SCFA which leads to an increase in cecal weight and decrease in cecal pH. Feeding rats with diets containing 0.3% grapefruit flavonoid extract, 5% or 10% Inulin, and a combination of both supplements resulted in a significant enlargement of the cecum compared to rats fed the control diet. Presence of dietary Inulin resulted in a larger cecal mass, over 4-fold higher than the control [51]. Inulin and soybean meal are both significant sources of fiber. Fermentation of fiber leads to the production of short-chain fatty acids such as butyric, propionic, acetic acid, and some gases. Butyrate is the preferred fuel for colonic mucosal cells [52]. Fiber can also increase fecal weight and speed intestinal transit.

Synergy1 singly and in combination with probiotics exhibited antitumorigenic activity against AOM-induced colon carcinogenesis which was explained by an increased production of cecal short-chain fatty acids (SCFAs) [53]. Prebiotics may modify the activity of beneficial intestinal microflora as a result of the lower cecal pH resulting in increased solubility of minerals such as calcium in acidic environment that may aid in bone and colonic health as well as increased isoflavone bioavailability [54].

Nutrients play a significant role in induction or inhibition of enzyme activities such as Phase 1 and Phase 2 enzymes that offer protection against xenobiotics by their detoxifying activity [55, 56]. Glutathione S-transferase (GST) is a phase 2 enzyme involved in conjugation and detoxification of potential carcinogens and its activity is enhanced by products of gut fermentation, such as SCFA [57–59]. Results from our study showed a significantly (*P* < .05) higher GST activity among treatment groups compared to the control. 

A similar study conducted using dietary fructo-oligosaccharides and isoflavone conjugates in ovariectomized mice showed increased *β*-glucosidase activity, equol production, and femoral bone mineral density representing contribution of FOS in increasing bioavailability of isoflavones [60]. Defatted Soybean meal flour and whole Soybean meal, good sources of isoflavones reduced the early stages of colon cancer [61]. 

 Results indicate that feeding Synergy1 in combination with Soybean meal had protective effects in reducing AOM-induced aberrant crypt foci in Fisher 344 male rats. One of the mechanisms that may explain the chemopreventive potential of Syn1 is the modulation of beneficial gut microflora (equol producing) by fermentation in the distal colon affecting soybean metabolism. Feeding Synergy1 and Soybean meal singly and in combination also enhanced the activity of antioxidative and detoxification enzymes which may have played a significant role in reducing AOM-induced ACF.

## 5. Conclusions

Feeding Synergy1 and Soybean in combination showed their potential health benefits in prevention of aberrant crypt foci in F344 male rats. However, ACF studies in rodents may be limited to evaluate the effects of functional foods over longer duration as advancement in cancer pathology is complex where ACF value to predict tumor outcome may decline [62]. Studies evaluating the combinational effects of Synergy1 and Soybean on end point tumor biomarkers, colon cancer specific genes, and signaling pathways may provide an insight on their synergism contributing to colon cancer prevention.

## Figures and Tables

**Table 1:** Composition of diets^a^ (AIN-93G).

Ingredients	Control (AIN-93G)	Syn1 (5%)	Syn1 (10%)	SM (5%)	SM (10%)	Syn1 + SM (5% + 5%)	Syn1 + SM (10% + 10%)	Syn1 + SM (5% + 10%)	Syn1 + SM (10% + 5%)
Syn1	0	50	100	0	0	50	100	50	100
SM	0	0	0	50	100	50	100	100	50
Corn starch	397.5	347.5	297.5	371.5	345.5	321.5	245.5	295.5	271.5
Casein	200	200	200	180	160	180	160	160	180
Fiber	50	50	50	46	42	46	42	42	46
Common Ingredients^b^	352.5	352.5	352.5	352.5	352.5	352.5	352.5	352.5	352.5

^a^Formulations of diets based on AIN-93G (Reeves et al., 1993^a,b^).

^b^CommonC ingredients (g): soybean oil: 70 g; mineral mix (AIN-93G): 35; vitamin mix: 10; L-cysteine: 3; choline bitartrate: 2.5.

Abbreviation used: Syn1: Synergy1, SM: Soybean meal, and AIN-93G: American Institute of Nutrition 93 Growth.

**Table 2:** Feed intake, weight gain, cecal weight, and cecal pH in rats fed Synergy1 and Soybean meal.

Groups	Feed intake g/day/rat	Weight gain (g)	Cecal weight (g)	Cecal wall (g)	Cecal pH
Control	17.53 ± 0.19^a^	275.67 ± 9.60^a^	2.58 ± 0.18^c^	1.02 ± 0.10^c^	7.40 ± 0.04^a^
Syn1 5%	16.77 ± 0.19^a^	278.33 ± 6.64^a^	3.04 ± 0.55^bc^	2.0 ± 0.30^b^	6.79 ± 0.90^b^
Syn1 10%	16.15 ± 0.47^a^	253.33 ± 10.90^a^	5.89 ± 0.13^a^	3.02 ± 0.06^a^	6.26 ± 1.00^bc^
SM 5%	16.59 ± 0.22^a^	254.00 ± 12.22^a^	2.91 ± 0.60^c^	1.51 ± 0.49^c^	7.00 ± 1.22^a^
SM 10%	17.29 ± 0.59^a^	257.33 ± 13.73^a^	2.91 ± 0.08^c^	1.32 ± 0.07^c^	7.06 ± 0.82^a^
Syn1 5% + SM 5%	16.91 ± 0.11^a^	266.00 ± 10.69^a^	3.70 ± 0.14^b^	2.06 ± 0.04^b^	6.22 ± 0.74^bc^
Syn1 10% + SM 10%	16.73 ± 0.16^a^	257.00 ± 9.07^a^	5.16 ± 0.78^a^	3.87 ± 0.19^a^	5.99 ± 0.20^c^
Syn1 5% + SM 10%	17.65 ± 0.07^a^	250.67 ± 2.02^a^	2.75 ± 0.21^c^	2.33 ± 0.19^b^	6.62 ± 1.11^b^
Syn1 10% + SM 5%	16.72 ± 0.81^a^	256.33 ± 15.37^a^	5.12 ± 0.71^ab^	3.76 ± 0.11^a^	6.10 ± 0.59^c^

Values are expressed as means ± SEM, *n* = 3.

^abc^Means in the same column with the same letter are not significantly different by Tukey's studentized range test (*P* ≤ .05).

Abbreviations used: Syn1: Synergy1 and SM: Soybean meal.

**Table 3:** Effect of Synergy1 and Soybean meal on incidence of aberrant crypt foci (ACF) in colon of Fisher 344 male rats.

Groups	Proximal Colon	Distal Colon	Total Colon
Control (AIN-93G)	49 ± 9.21^a^	101 ± 11.6^a^	150^a^
Syn1 5%	23 ± 1.5^b^	38 ± 4.1^b^	61^b^
Syn1 10%	17 ± 4.9^bc^	37 ± 12.4^b^	54^b^
SM 5%	24 ± 3.0^b^	48 ± 4.0^b^	72^b^
SM 10%	19 ± 2.8^bc^	44.5 ± 6.8^b^	63.5^b^
Syn1 5% + SM 5%	12 ± 5.5^bc^	32 ± 8.4^bc^	42^c^
Syn1 10% + SM 10%	9 ± 2.5^c^	32 ± 4.4^bc^	41^c^
Syn1 5% + SM 10%	4 ± 1.7^c^	51 ± 5.2^b^	49^bc^
Syn1 10% + SM 5%	7 ± 2.4^c^	27 ± 5.2^c^	34^c^

Values are expressed as means ± SEM; *n* = 3.

^abc^Means in the same column with the same letter are not significantly different by Tukey's studentized range test (*P* < .05).

Abbreviations used: Syn1: Synergy1 and SM: Soybean meal.

**Table 4:** Effect of Synergy1 and Soybean meal on total number of AOM-induced Crypts in Fisher 344 male Rats.

Groups	Proximal colon	Distal colon	Total colon
Control	141 ± 24.89^a^	310 ± 40.53^a^	444^a^
Syn1 5%	53 ± 5.1^bc^	136 ± 10.8^b^	189^bc^
Syn1 10%	46 ± 10.6^bc^	128 ± 28.3^b^	174^c^
SM 5%	72 ± 6.1^b^	152 ± 12.9^b^	224^b^
SM 10%	62 ± 8.8^b^	140 ± 26.2^b^	202^b^
Syn1 5% + SM 5%	31.5 ± 17.1^d^	106 ± 30.2^c^	137.5^d^
Syn1 10% + SM 10%	28 ± 7.5^d^	91 ± 10.3^c^	116^e^
Syn1 5% + SM 10%	22 ± 10.4^d^	121 ± 19.0^b^	144^d^
Syn1 10%+ SM 5%	27 ± 7.1^d^	72 ± 15.0^d^	99^e^

Values are expressed as means ± SEM; *n* = 3.

^abcde^Means in the same column with the same letter are not significantly different by Tukey's studentized range test (*P* < .05).

Abbreviations used: Syn1: Synergy1, SM: Soybean meal.

**Table 5:** Effect of Synergy1 and Soybean meal on glutathione S-transferase, catalase, and superoxide-dismutase activities and reduced glutathione level in Fisher 344 male rats.

Groups	GST (*μ*mol/mg)	GSH (mM)	CAT (*μ*mol/mL)	SOD (*μ*mol/mL)
Control (AIN-93G)	10.58 ± 0.32^b^	0.17 ± 0.13^b^	0.055 ± 1.50^b^	0.107 ± 0.008^b^
Syn1 5%	25.03 ± 0.81^a^	2.15 ± 0.12^a^	0.184 ± 0.02^a^	0.176 ± 0.013^a^
Syn1 10%	21.68 ± 1.85^a^	2.48 ± 0.13^a^	0.170 ± 0.01^a^	0.185 ± 0.001^a^
SM 5%	26.77 ± 2.56^a^	2.18 ± 0.28^a^	0.133 ± 0.23^a^	0.184 ± 0.008^a^
SM 10%	24.31 ± 0.38^a^	2.37 ± 0.78^a^	0.112 ± 0.01^a^	0.190 ± 0.009^a^
Syn1 5% +SM 5%	25.11 ± 2.28^a^	2.53 ± 0.27^a^	0.149 ± 0.04^a^	0.187 ± 0.007^a^
Syn1 10%+SM 10%	26.01 ± 4.04^a^	2.98 ± 0.16^a^	0.330 ± 0.19^a^	0.186 ± 0.005^a^
Syn1 5% +SM 10%	21.81 ± 0.71^a^	2.10 ± 0.19^a^	0.145 ± 0.02^a^	0.190 ± 0.006^a^
Syn1 10%+ SM 5%	25.28 ± 1.56^a^	2.13 ± 0.09^a^	0.127 ± 0.03^a^	0.186 ± 0.003^a^

Values are expressed as means ± SEM; *n* = 3.

^abc^Means in the same column with the same letter are not significantly different by Tukey's studentized range test (*P* < .05).

Abbreviations used: Syn1: Synergy1, SM: Soybean meal, GST: Glutathione S-transferase, GSH: reduced glutathione, CAT: catalase, and SOD: superoxide dismutase.

**Table 6:** Effect of control and treatment diets on Bone mineralization.

Groups	Ca (mg/g)	P (mg/g)	Mg (mg/g)	Fe (*μ*g/g)	Zn (*μ*g/g)
Control	268.5 ± 11.2^b^	122.4 ± 1.2^d^	2.2 ± 0.1^c^	53.1 ± 0.8^b^	163.9 ± 24.8^b^
Syn1 5%	373.1 ± 2.7^a^	145.7 ± 1.3^bc^	3.1 ± 0.05^ab^	63.0 ± 3.3^ab^	218.6 ± 3.6^ab^
Syn1 10%	374.6 ± 5.7^a^	147.3 ± 2.6^bc^	3.1 ± 0.06^ab^	65.6 ± 0.5^ab^	231.4 ± 6.0^a^
SM 5%	393.2 ± 18.9^a^	147.4 ± 3.0^bc^	3.1 ± 0.03^ab^	63.9 ± 2.5^ab^	227.4 ± 19.3^a^
SM 10%	353.3 ± 12.8^a^	142.6 ± 1.5^c^	2.8 ± 0.08^b^	58.4 ± 4.0^ab^	214.0 ± 11.9^ab^
Syn 1 + SM 5%	416.3 ± 6.8^a^	152.4 ± 1.2^b^	3.3 ± 0.1^a^	67.4 ± 4.6^a^	249.6 ± 5.3^a^
**Syn 1 + SM 10%**	417.6 ± 32.4^**a**^	166.2 ± 1.3^**a**^	3.4 ± 0.1^**a**^	70.7 ± 2.9^**a**^	250.3 ± 5.0^**a**^
Syn1 5% + SM 10%	394.8 ± 2.8^a^	153.9 ± 1.1^b^	3.3 ± 0.09^a^	66.1 ± 2.4^ab^	246.2 ± 6.6^a^
Syn1 10% + SM 5%	396.3 ± 6.5^a^	152.1 ± 2.2^b^	3.2 ± 0.07a^b^	66.6 ± 1.5^ab^	236.2 ± 1.7^a^

Values are means ± SEM; *n* = 3.

^abcd^Means in a column with same superscripts do not significantly differ (*P* < .05).

Abbreviations: Syn1: Synergy1; SM: Soybean meal; Ca: calcium; P: phosphorous, Mg: magnesium; Fe: iron; Zn: zinc.
